# Identification and Experimental Validation of Biomarkers Associated With Mitochondria and Macrophage Polarization in Sepsis

**DOI:** 10.1155/emmi/8755175

**Published:** 2025-05-19

**Authors:** Liping She, Xiaojing Deng, Yeping Bian, Hui Cheng, Jian Xu

**Affiliations:** Department of Intensive Care Unit, Geriatric Hospital of Nanjing Medical University, Nanjing, China

**Keywords:** biomarkers, macrophage polarization, mitochondria, sepsis

## Abstract

**Background:** Sepsis is a common and serious condition, where mitochondria and macrophage polarization play a crucial role. Therefore, this study aimed to identify and validate biomarkers for sepsis associated with mitochondria-related genes (MCRGs) and macrophage polarization–related genes (MPRGs), providing new targets and strategies for therapeutic intervention.

**Methods:** This study utilized the GSE95233 and GSE28750 datasets. Initially, intersection genes were identified by overlapping MCRGs and the results from differential expression analysis and weighted gene co-expression network analysis (WGCNA). Biomarkers were identified through machine learning and gene expression analysis. A nomogram was developed and evaluated based on these biomarkers. Finally, functional enrichment, immune infiltration, and reverse transcription quantitative polymerase chain reaction (RT-qPCR) analyses were conducted to further elucidate the biological mechanisms underlying sepsis.

**Results:** The study identified YME1L1, ECHDC3, THEM4, and COQ10A as biomarkers for sepsis. Among them, YME1L1, THEM4, and COQ10A showed significantly lower expression levels in sepsis samples, while ECHDC3 exhibited markedly higher expression. Notably, RT-qPCR analysis confirmed that YME1L1, THEM4, and COQ10A exhibited significantly lower expression levels in sepsis samples. A nomogram based on these biomarkers was developed and validated, effectively predicting sepsis risk. Enrichment analysis indicated that the biomarkers were co-enriched in the oxidative phosphorylation pathway. Additionally, 13 significantly different immune cell types were identified between sepsis and control samples. Biomarker association analysis revealed that CD8 T cells had the strongest positive correlation with YME1L1 (cor = 0.84, *p* < 0.05) and the strongest negative correlation with ECHDC3 (cor = −0.76, *p* < 0.05), suggesting their potential role in the disease mechanism.

**Conclusion:** In this study, YME1L1, ECHDC3, THEM4, and COQ10A were identified as biomarkers for sepsis, with their expression levels validated in clinical samples. These findings provided a promising theoretical foundation for the development of targeted treatments for sepsis.

## 1. Introduction

Sepsis is a life-threatening condition response to infection injures which is defined as the systemic inflammatory response syndrome [1, 2]. It is deteriorated by the inappropriate immune response resulting in high morbidity and mortality [3]. In the global world, more than 19 million severe sepsis cases occur every year, with at least five million sepsis and sepsis-related deaths [4]. The management of sepsis and sepsis shock includes anti-infective therapy, maintenance of hemodynamic stability, and so on [5]. However, the diagnosis of sepsis or septic shock is absence of a single indicator or measurement method. Biomarkers are considered as indicators of infection or treatment and help clinicians to estimate patient risk, which have been studied in prediction of sepsis, diagnosis of sepsis, assessment of sepsis response to therapy, and biomarker-guided antibiotic therapy [6].

Mitochondria are considered as important organelle in eukaryotic cells, which is closely related to some cellular biological processes including energy conversion, redox equilibrium, and so on [7]. Mitochondrial dysfunction is important in the progression of sepsis, which is pivotal in sepsis progression and the ensuing multiorgan dysfunction [8]. Macrophages are important immune cells related to the immune mechanisms of sepsis, which can differentiate into distinct subtypes at different stages of the inflammatory response. The polarization of macrophages results from inflammation, and controlling macrophage polarization is a potential strategy for sepsis management [9]. Mitochondrial damage–associated molecular patterns (mtDAMPs) can stimulate the immune system [10]. The mitochondrial release induced by mtDAMPs can increase the ATP level, and extracellular ATP relies on macrophage P2X7 purinergic receptors, enhancing intracellular bacterial killing, to be against sepsis [11]. Exploring the sepsis associated with mitochondria-related genes (MCRGs) and macrophage polarization–related genes (MPRGs) can help us further understand the mechanism of sepsis and provide a new target for the treatment of sepsis.

This study used data from the Gene Expression Omnibus (GEO) database and conducted differential expression analysis, weighted gene co-expression network analysis (WGCNA), machine learning, and gene expression analysis to identify biomarkers related to mitochondria and macrophage polarization in sepsis. These biomarkers were validated by reverse transcription quantitative polymerase chain reaction (RT-qPCR) analysis in clinical samples. Furthermore, we conducted functional exploration, built a pathway map, and performed immune infiltration analysis to further explore the potential mechanisms of these biomarkers. All these analyses provide new insights into the diagnosis and treatment of sepsis.

## 2. Materials and Methods

### 2.1. Data Collection

Transcriptome data for sepsis were obtained from the GEO database (https://www.ncbi.nlm.nih.gov/geo/). Specifically, GSE95233 dataset (GPL570 platform) included 51 blood samples from sepsis patients and 22 control blood samples, all collected on day 1. Samples from days 2 and 3 were not included, and instances of sepsis in nonsurvivors were removed from the dataset (Supporting Table S1). Additionally, the GSE28750 dataset (GPL570 platform) comprised 10 sepsis and 20 control whole blood samples (Supporting Table S2). Furthermore, 1136 MCRGs were retrieved from the MitoCarta3.0 database (https://www.broadinstitute.org/mitocarta/mitocarta30-inventory-mammalian-mitochondrial-proteins-and-pathways) [12], and 35 MPRGs were acquired from molecular signatures database (MSigDB, https://www.gsea-msigdb.org/gsea/msigdb) [13] (Supporting Table S3).

### 2.2. Differential Expression Analysis

Differentially expressed genes (DEGs) between sepsis patients and control samples in the GSE95233 dataset were ascertained by “limma” (v 3.58.1) [14], with thresholds set at |log_2_ fold-change (FC)| > 1 and *p* < 0.05. The DEGs were visualized using a volcano plot and heatmap. Specifically, the volcano plot was generated with the “ggplot2” package (v 3.4.2) [15], while the heatmap displayed the top 10 up- and downregulated genes ranked by |log_2_ FC| from high to low, created using the “ComplexHeatmap” package (v 2.16.0) [15].

### 2.3. WGCNA

In this study, MPRGs were utilized to calculate the MPRG score using single-sample gene set enrichment analysis (ssGSEA) algorithm from the “GSVA” package (v 1.50.0) [16]. In the GSE95233 dataset, the differences in MPRG score between sepsis patients and control samples were compared using the Wilcoxon test (*p* < 0.05). Subsequently, in the GSE95233 dataset, WGCNA was conducted to identify the key module most associated with the MPRG score, employing the “WGCNA” package (v 1.72-5) [17]. Initial hierarchical clustering analysis was performed using the “GoodSamplesGenes” function to cluster all samples and remove outliers. An optimal soft threshold (power) was then determined using the “pickSoftThreshold” function, targeting a scale-free topology with an *R*^2^ exceeding 0.9 while ensuring the mean connectivity remained close to 0. A hierarchical clustering tree was constructed with a minModuleSize set to 150 and a mergeCutHeight of 0.4, resulting in distinct gene modules represented by different colors. Following module identification, Pearson correlation coefficients were calculated between the MPRG score and each gene module (|cor| > 0.3, *p* < 0.05). Modules exhibiting the highest correlations with the MPRG score were designated as key modules. The genes within these key modules were recorded as key module genes for further detailed analysis.

### 2.4. Identification and Function Analysis of Intersection Genes

Intersection genes were identified by overlapping DEGs, key module genes, and MCRGs using the “VennDiagram” package (v 1.2.3) [18]. Subsequently, GO and KEGG analyses were conducted to explore the biological functions and pathways associated with these intersection genes, utilizing the “clusterProfiler” package (v 4.8.2) [19], with a significance threshold set at *p* < 0.05. Additionally, to examine protein interactions among the intersection genes, a protein–protein interaction (PPI) network was constructed using STRING (https://string-db.org/), applying a confidence score threshold of 0.15. After removing outlier genes, the resulting network was visualized using Cytoscape (v 3.7.1) [20].

### 2.5. Determination of Biomarkers

Based on the intersection genes, the least absolute shrinkage and selection operator (LASSO) method was applied to identify feature genes1 within the GSE95233 dataset. Specifically, LASSO analysis was conducted using the “glmnet” package (v 4.1-8) [21], with results confirmed at the minimum lambda value. At the same time, the Boruta analysis was carried out using the Boruta package (v 8.0.0) [22]. Based on the importance ranking of the genes, feature genes2 was identified. After that, the intersection of feature genes1 and feature genes1 was taken to obtain the key genes. The VennDiagram package (v 1.2.3) was utilized to draw the Venn diagram. Following this, gene expression analyses of the identified key genes were performed in both the GSE95233 and GSE28750 datasets using Wilcoxon test (*p* < 0.05). The results were displayed in boxplot using “ggplot2.” Genes that demonstrated significant differential expression between sepsis patients and control samples (*p* < 0.05) and exhibited consistent expression trends across both datasets were classified as biomarkers for further investigation.

### 2.6. Construction and Validation of Nomogram

Based on the identified biomarkers, a nomogram was developed to predict the risk of sepsis using the “regplot” package (v 1.1) [23]. To assess the accuracy of the nomogram, calibration curves and decision curves were generated. Notably, the decision curves were plotted using “rmda” package (v 1.6) (https://CRAN.R-project.org/package=rmda). Additionally, a receiver operating characteristic (ROC) curve was created using the “pROC” package (v 1.18.5) [24] to evaluate the model's performance.

### 2.7. Function Analysis of Biomarkers

To further elucidate the biological functions and signaling pathways associated with the biomarkers, Spearman correlations between the biomarkers and other genes were calculated and ranked (from high to low) in the GSE95233 dataset using the “corrplot” package (v 0.92) [25]. The “C2: KEGG gene sets” were obtained from the MSigDB database as the background set. GSEA was then performed using the “clusterProfiler” package to enrich the ranked genes within this background set (*p* < 0.05). Additionally, GeneMANIA (https://genemania.org/) was employed to identify genes functionally related to the biomarkers.

### 2.8. Immune Infiltration Analysis

To investigate immune cell infiltration in sepsis patients compared to control samples from the GSE95233 dataset, an immune infiltration analysis was performed using the CIBERSORT algorithm (v 2.1.0) [26]. This method allowed us to calculate infiltration scores for 22 distinct immune cell types [27] across the samples. Subsequently, the differences in infiltration scores between sepsis patients and control samples were assessed using the Wilcoxon test, and the results were presented using “ggplot2.” Immune cell types with significant differential infiltration (*p* < 0.05) were selected for further analysis. Following this, Spearman correlation analysis was conducted using the “psych” package (v 2.4.3) [28] to explore relationships among the differentially infiltrated immune cells, the associations among biomarkers, and the correlations between these immune cells and biomarkers (|cor| > 0.30, *p* < 0.05).

### 2.9. RT-qPCR

To validate the expression of biomarkers in clinical samples, RT-qPCR analysis was performed. Specifically, a total of 10 blood samples (5 controls and 5 cases) were acquired from the clinic in Geriatric Hospital of Nanjing Medical University. The study was conducted in accordance with the Declaration of Helsinki and approved by the Ethics Committee of Geriatric Hospital of Nanjing Medical University (approval number: 2024-026-1). All participants gave informed consent. Clinical information for all samples is provided in Supporting Table S4. Total RNA from the 10 samples was extracted with the TRIzol reagent (Ambion, USA) according to the manufacturer's protocol. Then the RNA concentration was tasted using NanoPhotometer N50. The cDNA was synthesized by reverse transcription using the SureScript-First-Strand-cDNA-Synthesis-Kit, and the reverse transcription was performed with the S1000 Thermal Cycler (Bio-Rad, USA). The sequences of all primers can be found in Supporting Table S5. The qPCR assay was performed with CFX Connect Real-time Quantitative Fluorescence PCR Instrument (Bio-Rad, USA) (predenaturation at 95°C for 1 min, denaturation at 95°C for 20 s, annealing at 55°C for 20 s, and extension at 72°C for 30 s, a total of 40 cycles). The relative quantification of mRNAs was calculated using the 2^−ΔΔCT^ method. The results from the RT-qPCR were exported to Excel and then imported into GraphPad Prism 5 for statistical analysis and visualization (*p* < 0.05).

### 2.10. Statistical Analysis

R (v 4.3.3) was utilized to conduct statistical analysis. Difference analysis between groups was executed via the Wilcoxon test (*p* < 0.05). Notably, ^∗∗∗∗^represented *p* < 0.0001, ^∗∗∗^represented *p* < 0.001, ^∗∗^represented *p* < 0.01, ^∗^represented *p* < 0.05, and ns represented *p* > 0.05.

## 3. Results

### 3.1. Intersection Genes Were Identified

**Figure 1:**
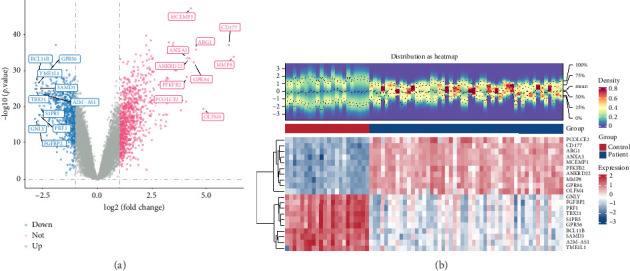
Plot of results of differential expression analysis. (a) DEGs Volcano map. Horizontal coordinates multiplicity of differences. Vertical coordinates represented −log10 (pval). Each point in the graph represented a gene; blue points on the left indicated down-regulated genes and red points on the right indicated up-regulated genes. (b) DEGs Heatmap (TOP 10, each small square represented each gene, and its color indicated the level of expression of that gene, the higher the expression the darker the color (red is high expression, blue is low expression).

**Figure 2:**
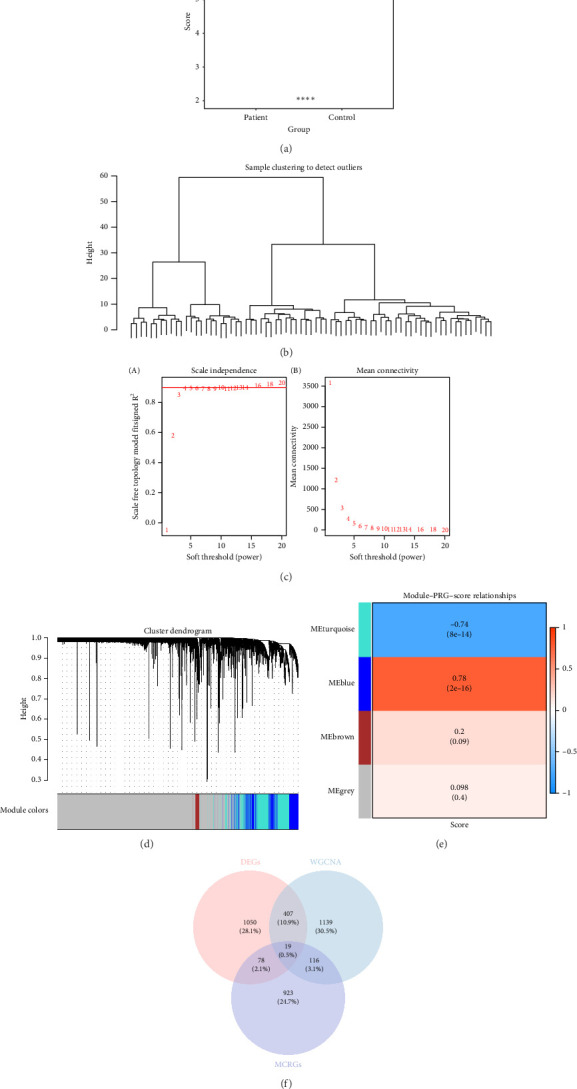
Results of WGCNA identification of modular genes related to MP. (a) Box plots of MPRGs scores for disease and control samples, green boxes were patients, orange boxes were control samples. (b) Clustering plot of all samples in the training set, each vertical line at the bottom represented a sample, and the upper split vertical lines represented sample clustering. (c) Scale-free networks with soft threshold screening, (left) scale-free fit index, (right) average connectivity. (d) Systematic clustering tree between genes, module colors represented different gene modules or gene clusters. (e) Heatmap of gene module correlation with MPRGs scores, a total of 4 modules were related to MPRGs scores. (f) Candidate gene Venn diagram, pink circles represented DEGs, blue circles represent MP module genes, purple circles represent MCRGs.

Through differential expression analysis, a total of 1554 DEGs were identified. Among these, 881 genes were upregulated and 673 genes were downregulated in sepsis patients (Figures 1(a) and 1(b)). Additionally, the MPRG score was significantly higher in control samples compared to sepsis patients (*p* < 0.05) (Figure 2(a)). In the WGCNA of the GSE95233 dataset, no outlier samples were detected (Figure 2(b)). The optimal power value was determined to be 16, as it exceeded the threshold indicated by the red line (*R*^2^ = 0.9) and the mean connectivity approached zero (Figure 2(c)). Utilizing the hierarchical clustering tree, similar modules were merged, resulting in the identification of four gene modules, including a gray module for unclassified genes, each represented by a distinct color (Figure 2(d)). Notably, the MEblue module exhibited the strongest correlation with the MPRG score (cor = 0.78, *p* = 2 × 10^−16^) (Figure 2(e)). Consequently, the 1681 genes within the MEblue module were considered as key module genes. By intersecting the 1554 DEGs, 1681 key module genes, and 1136 MCRGs, a total of 19 intersection genes were identified (Figure 2(f)). In summary, a series of analyses successfully identified 19 intersection genes associated with sepsis.

### 3.2. Functions of Intersection Genes Were Explored

**Figure 3:**
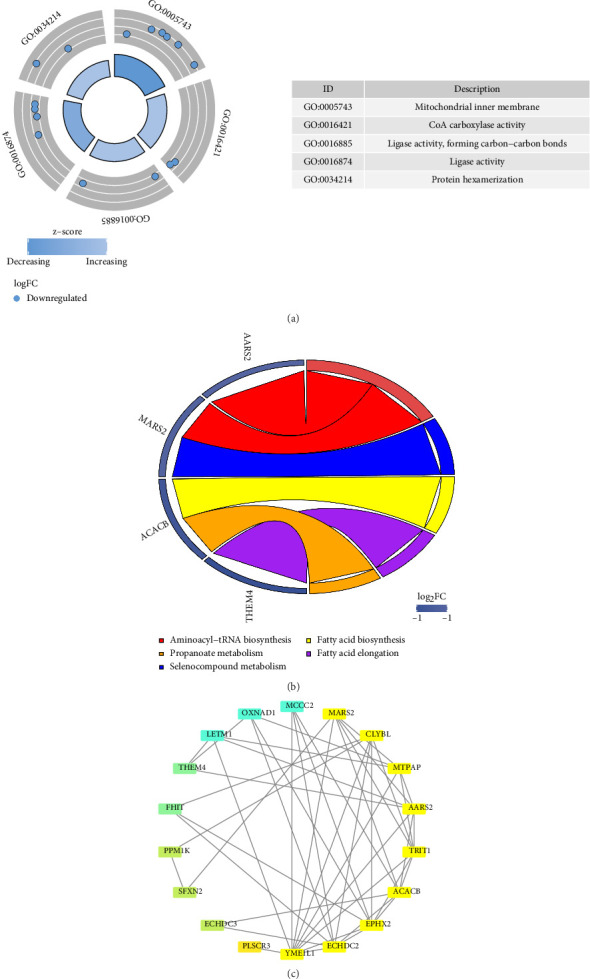
Functional analysis of intersection genes. (a) GO enrichment results; the blue dots represented downward adjustments. (b) KEGG enrichment results; 5 different colors for 5 different enrichment results. (c) Candidate gene PPI analysis result map, where the lines represented their interactions and the thickness represented the degree of their bonding.

Functional enrichment analysis showed that the 19 intersection genes were significantly enriched in 44 GO terms (30 MFs, 12 BPs, and 2 CCs) and seven KEGG pathways. The top five GO terms and KEGG pathways, ranked by *p* value from low to high, were presented. For instance, significant GO terms included “protein hexamerization” (BP), “mitochondrial inner membrane” (CC), and “CoA carboxylase activity” (MF) (Figure 3(a), Supporting Table S6). Detailedly, the identified KEGG pathways encompassed “aminoacyl-tRNA biosynthesis,” “propanoate metabolism,” “selenocompound metabolism,” “fatty acid biosynthesis,” “fatty acid elongation,” “pyruvate metabolism,” and “valine, leucine, and isoleucine degradation” (Figure 3(b), Supporting Table S7). Besides, removing one outlier gene resulted in a PPI network composed of 18 genes and 45 interactions (Figure 3(c)). Notably, YME1L1 demonstrated close interactions with several genes, including LETM1 and TRIT1. In summary, these intersection genes were involved in multiple functions and pathways, highlighting their potential roles in sepsis.

### 3.3. The Four Biomarkers of Sepsis Were Ascertained

**Figure 4:**
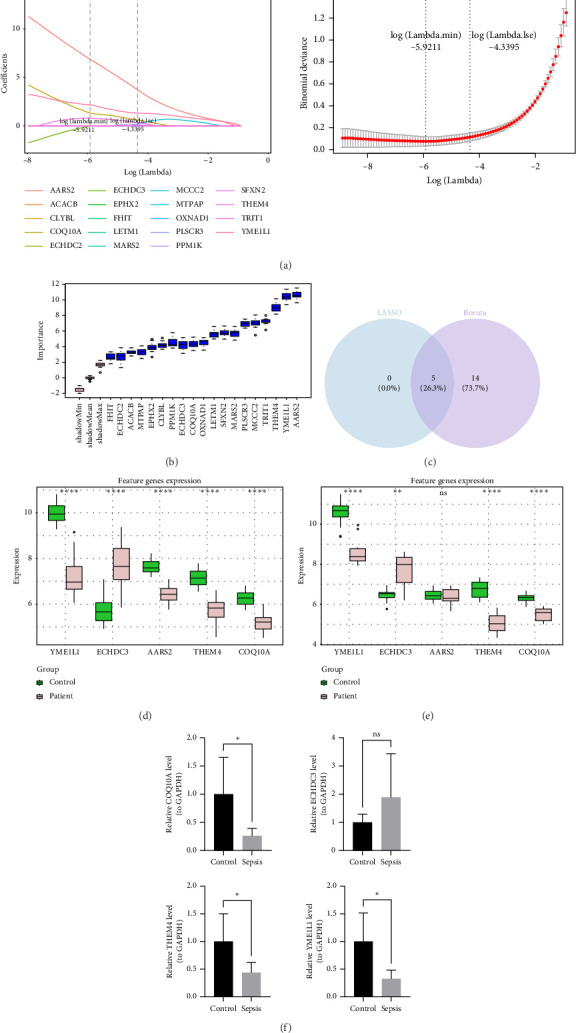
Identification of 4 biomarkers of sepsis. (a) LASSO regularized path diagrams, the horizontal coordinate represents the value of the regularization parameter and the vertical coordinate represented the absolute or scaled value of the characteristic coefficients. And LASSO coefficient path diagrams, the horizontal coordinates represent the values of the regularization parameters, the vertical coordinates represented the performance metrics of the model. (b) Plot of the results of raw letter analysis based on Boruta's algorithm. Horizontal coordinates were gene names and vertical coordinates were feature importance scores calculated by Boruta's algorithm. (c) Venn diagram of the intersection of the LASSO and Boruta feature genes. The overlapping part represented the number of key genes. (d) Biomarker expression levels in the training set, pink boxes were septic patients in the training set, green boxes were control samples in the training set. ^∗∗∗∗^*p* < 0.0001. (e) Expression levels of biomarkers in the validation set.^∗∗^*p* < 0.01; ^∗∗∗∗^*p* < 0.0001; ns, not significance. (f) RT-PCR results for YME1L1, THEM4, COQ10A, and ECHDC3. ^∗^*p* < 0.05; ns, not significance.

From an initial set of 19 intersection genes, the LASSO method identified five feature genes1 with a log(lambda.min) of −5.9211 (Figure 4(a)). Meanwhile, 14 feature genes2 were determined in the Boruta analysis (Figure 4(b)). Then, five key genes were acquired after the overlap of feature genes1 and 14 feature genes2 (Figure 4(c)). Subsequent gene expression analysis revealed that YME1L1, THEM4, and COQ10A exhibited significantly lower expression levels in sepsis patient samples from both the GSE95233 and GSE28750 datasets, while ECHDC3 demonstrated markedly higher expression in sepsis patients (*p* < 0.05) (Figures 4(d) and 4(e)). Consequently, YME1L1, THEM4, COQ10A, and ECHDC3 were identified as biomarkers for sepsis. RT-qPCR results showed YME1L1, THEM4, and COQ10A were obviously lower expression in sepsis samples (*p* < 0.05), while ECHDC3 did not significant differences between sepsis and control samples (Figure 4(f)). The results emphasized the expression patterns of these biomarkers in clinical samples.

### 3.4. A Good Nomogram Was Constructed

**Figure 5:**
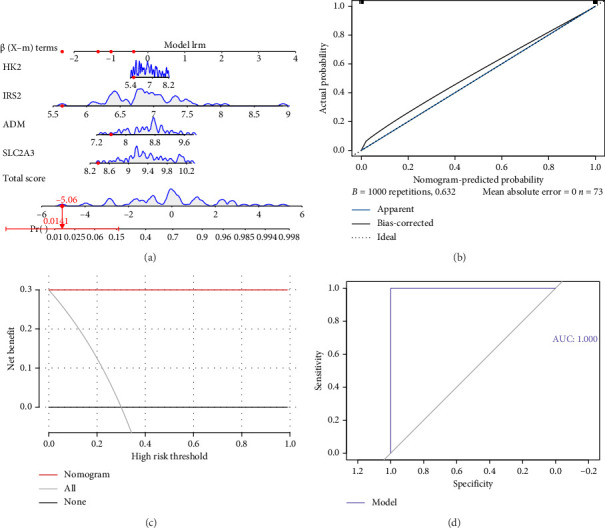
Construction of biomarker nomogram. (a) Nomogram of the training set; the scale represented the range of values that could be taken for the biomarker, while the length of the line reflected the magnitude of the gene's contribution to the disease. (b) Calibration curves for training set nomogram, where the horizontal coordinate was the predicted event rate and the vertical coordinate is the observed actual event rate. (c) Decision curves for the training set; the horizontal coordinate was the threshold probability and the vertical coordinate was the net benefit. (d) ROC curve of the training set.

Based on the four biomarkers, a nomogram was developed (Figure 5(a)). The nomogram indicated that higher total points were associated with an increased risk of sepsis. The calibration curve confirmed that the model provided accurate predictions (Figure 5(b)). Additionally, decision curve analysis (DCA) demonstrated that the model's net benefit was superior to that of any single factor (Figure 5(c)). The ROC curve revealed an AUC of 1.000 for the nomogram, indicating its exceptional predictive performance (Figure 5(d)). Overall, these results underscored the nomogram's robust efficacy in predicting sepsis.

### 3.5. Functions and Pathways Associated With Biomarkers Were Investigated

**Figure 6:**
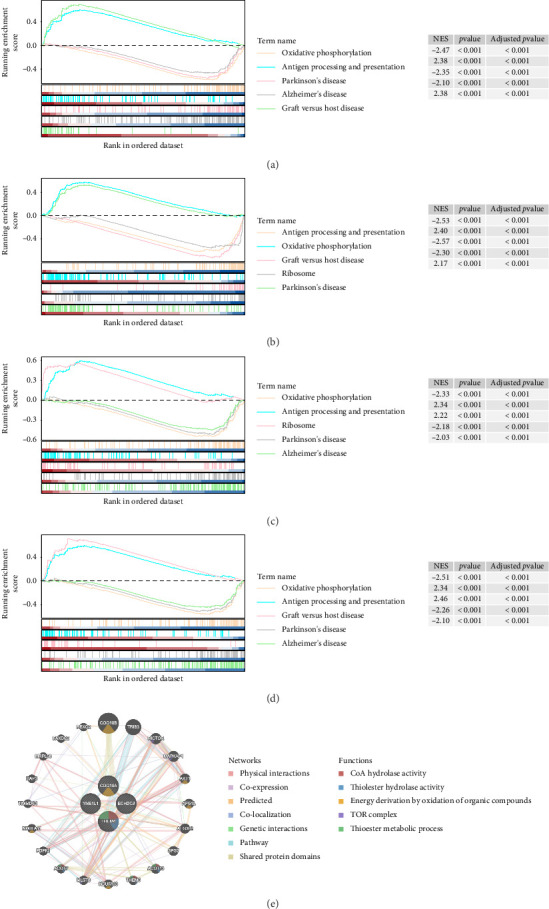
GSEA enrichment analysis diagram. (a) GSEA enrichment analysis of YME1L1 in the training set; the score at the highest/lowest point was the corresponding ES value for that pathway, each vertical line represented a gene, and the color from red to blue indicates a positive to negative correlation. (b) GSEA enrichment analysis of ECHDC3 in the training set. (c) GSEA enrichment analysis of THEM4 in the training set. (d) GSEA enrichment analysis of COQ10A in the training set. (e) GeneMANIA results; different colored lines represent different modes of action, and different colors in the gene circle represented correlations with 5 different functions.

YME1L1, ECHDC3, THEM4, and COQ10A were obviously enriched in 49, 51, 53, and 52 KEGG pathways (Supporting Table S8). Notably, GSEA revealed significant co-enrichment of the four biomarkers in pathways related to “oxidative phosphorylation,” “Parkinson's disease,” and “antigen processing and presentation” (Figures 6(a), 6(b), 6(c), and 6(d)). Using GeneMANIA, 20 functionally related genes associated with the biomarkers were identified. Notable interactions included COQ10A with COQ10B, ECHDC3 with AKT1, THEM4 with MLST8, and YME1L1 with NDUFA11, highlighting their roles in processes such as “CoA hydrolase activity,” “thiolester hydrolase activity,” and the “TOR complex” (Figure 6(e)). These findings emphasized the potential involvement of these biomarkers in critical biological pathways and processes, offering valuable insights into their roles in the progression of sepsis.

### 3.6. The Immune Infiltration Differences Were Explored

**Figure 7:**
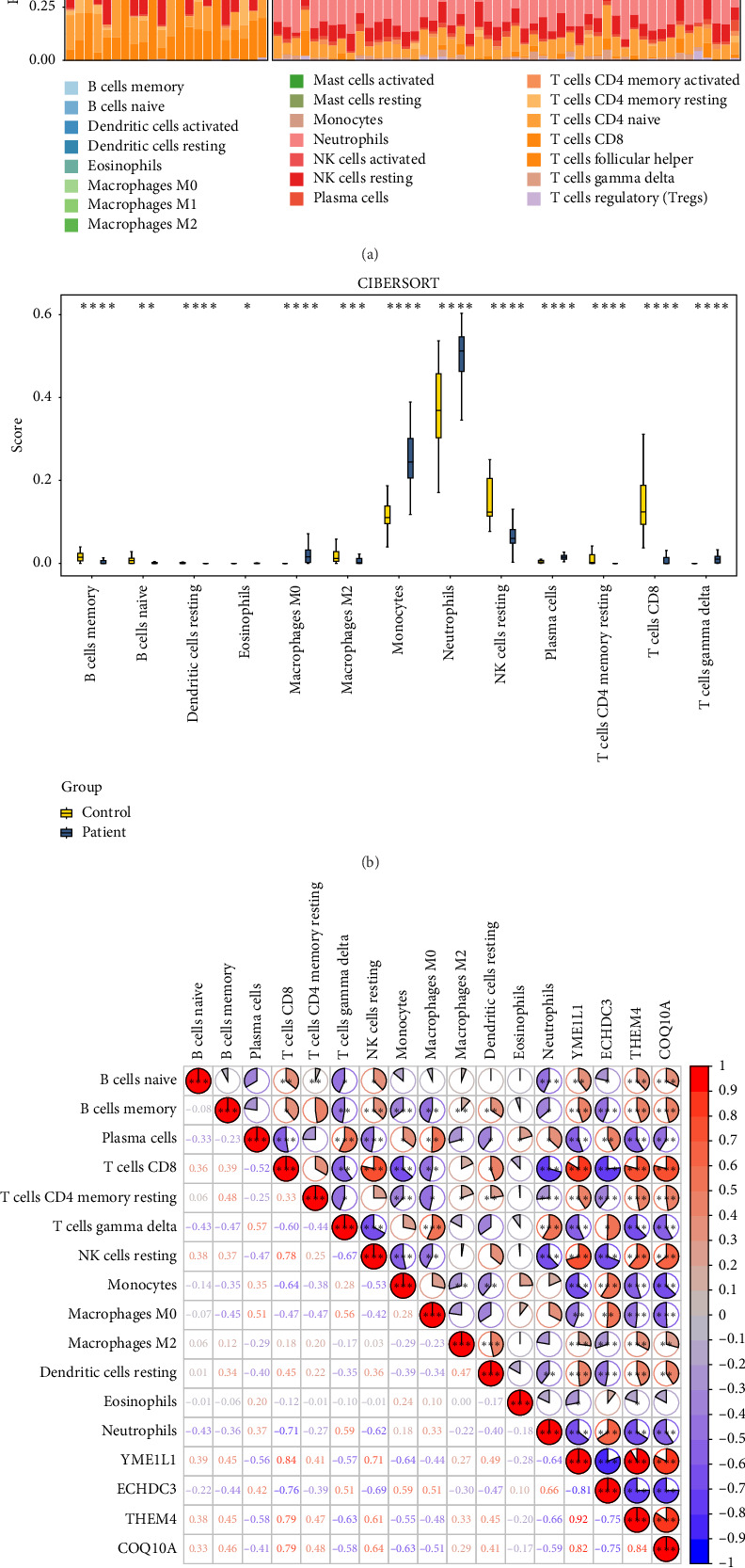
Immunocyte infiltration analysis result chart. (a) Distribution of immune cells in training centers; the stacked plot showed the proportion of 22 immune cells in each sample. (b) Box plot of immune cell differences between control and disease groups in the training set; the horizontal coordinate was the cell type and the vertical coordinate is the immune infiltration score. (c) Correlation analysis between training set difference immune cells and biomarkers, where red represented positive correlation and blue represented negative correlation.

Neutrophils exhibited a high proportion of infiltration in both sepsis patients and control samples (Figure 7(a)). Analysis revealed significant differences in the infiltration levels of 13 immune cell types between sepsis patients and controls (*p* < 0.05) (Figure 7(b)). Specifically, memory B cells and naive B cells were more prevalent in control samples, while monocytes and neutrophils showed higher infiltration in sepsis patients. Correlation analysis among these differential immune cells demonstrated a strong negative association between CD8 T cells and neutrophils (cor = −0.71, *p* < 0.05) and a robust positive correlation between CD8 T cells and resting NK cells (cor = 0.78, *p* < 0.05) (Figure 7(c)). Moreover, biomarker association analysis revealed that CD8 T cells had the strongest positive correlation with YME1L1 (cor = 0.84, *p* < 0.05) and the strongest negative correlation with ECHDC3 (cor = −0.76, *p* < 0.05) (Figure 7(c)). Besides, YME1L1 showed a strong positive correlation with THEM4 (cor = 0.92, *p* < 0.05) and a notable negative correlation with ECHDC3 (cor = −0.81, *p* < 0.05) (Figure 7(c)). These findings highlighted the distinct immune landscape in sepsis and underscored the critical relationships between specific immune cells and biomarkers, providing insights into potential therapeutic targets for managing sepsis.

## 4. Discussion

Sepsis is the leading cause of death from infection in the world, which should be early diagnosed for the mortality rate of 40% or more [29]. However, the lack of diagnostic methods for sepsis results in challenges in its diagnosis. Mitochondrial damage plays a crucial role in the sepsis progression. Many factors contribute to mitochondrial impairment during sepsis including generation of reactive oxygen species, inhibition of mitophagy, and change of mitochondrial dynamics [30]. Macrophages play an important role in rebuilding immune balance by modulating the inflammatory response in patients with sepsis [31]. The mitochondrial release can increase the ATP and enhance the intracellular bacterial killing of macrophage [11]. However, the molecular mechanism between the two and sepsis is unclear. Therefore, machine learning and gene expression analysis were utilized in this study to identify YME1L1, ECHDC3, THEM4, and COQ10A as biomarkers for sepsis in our study. Among them, YME1L1, THEM4, and COQ10A showed significantly lower expression levels in sepsis samples, while ECHDC3 exhibited markedly higher expression. Biomarkers such as GSEA and immune infiltration were also analyzed. These findings provide the targets for the diagnosis and treatment of sepsis.

The member of the AAA family of ATPases, YME1L1, is a nuclear genome-encoded ATP-dependent metalloprotease which was embedded in the inner mitochondrial membrane (IM) [32]. YME1L1 is an upstream regulatory molecule of optic atrophy protein 1 (OPA1). Because of the importance of YME1L1 for mitochondrial functionality in humans, YME1L1 mutations may result in mitochondriopathy [33]. Previous study has demonstrated that LPS-induced mitochondrial damage can be alleviated by promoting OPA1-mediated mitochondrial fusion through YME1L1 [34]. Another study indicates that tumor suppressor KIF1B *β* regulates the structural and functional dynamics of mitochondria with YME1L1 and plays an important role in apoptosis mediated by mitochondria [35]. The cellular injury and apoptosis were thought to result from the abnormal balance of fission and fusion of mitochondria through the animal model of sepsis [36]. This study has found that YME1L1 showed significantly lower expression levels in sepsis samples. The low expression of YME1L1 may lead to mitochondrial dysfunctionality in patients with sepsis. These studies demonstrated that YME1L1 may play a role in sepsis by influencing mitochondrial function.

Enoyl-CoA hydratase domain containing 3 (ECHDC3) is active in the mitochondrion and participates in fatty acid biosynthesis and lipid metabolism which is widely expressed in adipocytes [37]. ECHDC3 may play an important role in the regulation of intracellular energy balance and metabolic state and has a certain impact on the response to environmental changes and cellular stress. Sharma et al. reported that 691 DEGs were identified in ECHDC3-knockdown adipocytes. The genes were enriched in known insulin resistance genes [38]. Compared to the patients without lesion, ECHDC3 mRNA expression was 1.2-fold higher in low lesion patients with the serum levels of oleic acid and total monounsaturated fatty acids elevated [39]. Changes in lipid metabolism and activation of lipid signaling pathways are components of the complex environment underlying the pathological and physiological sequelae of sepsis [40]. ECHDC3 exhibited markedly higher expression in sepsis patients in our study, which destroyed the fatty acid biosynthesis and lipid metabolism. This may verify that ECHDC3 can prompt the development of sepsis by regulating lipid metabolism.

Thioesterase superfamily member 4 (THEM4) as one of the Akt kinase-binding proteins is a member of the thioesterase superfamily. Some studies have demonstrated that THEM4 binds to Akt to regulate its phosphorylation on the mechanism of cancers and other diseases [41, 42]. Increased PD-L1 expression was found to trigger AKT phosphorylation, which in turn increased mortality during clinical and experimental sepsis. This suggests that THEM4 may play a role in sepsis by regulating phosphorylation.

COQ10A is essential for the biosynthetic function of COQ which is primarily expressed in skeletal muscle cells and myocardial cells. Coenzyme Q (CoQ) is a redox-active lipid which is necessary for proton transport and respiratory electron during cellular energy metabolism. COQ10A, putative regulatory circuit of autosomal genes relevant with mitochondrial respiratory chain and heart development, was revealed by competing endogenous RNA network analysis [43]. Therefore, the dysfunction of oxidative phosphorylation might result from the inhibition of COQ10A expression [44]. Mitochondrial oxidative phosphorylation is necessary for the production of ATP under aerobic conditions. Sepsis severely affects the energy supply of the cell, and energy demand is difficult to quantify [45]. We identified COQ10A as biomarkers for sepsis, which showed significantly lower expression levels in patients with sepsis. Downregulation of COQ10A may prompt the development of sepsis by inhibition of oxidative phosphorylation.

This research predicted sepsis risk by a nomogram which was developed and validated based on biomarkers. Enrichment analysis indicated that the biomarkers were co-enriched in the oxidative phosphorylation pathway. It has elucidated the relationship between the oxidative phosphorylation pathway and sepsis, such as sepsis-induced impairment of mitochondrial oxidative phosphorylation in cardiomyocytes [46]. Oxidative phosphorylation primarily occurs in the mitochondria, while processes such as the assembly of antigenic peptides with major histocompatibility complex class I (MHC-I) molecules during antigen processing and presentation take place in the endoplasmic reticulum. Endoplasmic reticulum–mitochondria contacts are crucial for regulating lipid transport, synthesis, and metabolism [47]. Therefore, this interaction between oxidative phosphorylation and antigen processing/presentation may play a pivotal role in the development of sepsis. The mitochondrial remodeling is related to various energy metabolism pathways including fatty acid catabolism, the electron transport chain, and oxidative metabolism of carbohydrate. The oxidative metabolism mediated by the oxidative phosphorylation pathway through the mitochondria plays an important role in sepsis. This suggests that biomarkers may play an important role in sepsis through the oxidative phosphorylation pathway.

Additionally, we have verified 13 significantly different immune cell types between sepsis and control samples. Biomarker association analysis revealed that CD8 T cells had the strongest positive correlation with YME1L1 and the strongest negative correlation with ECHDC3. CD8+ T cells are effector cells of the adaptive immune response. Sepsis changes host capacity to respond to re-infection because of long-term alterations in memory CD8+ T cell phenotype, protective function, and localization [48]. Alves et al. [49] reported that the proteins extracted from the monocytes of individuals with septic shock are involved in monocyte reprogramming, immune dysfunction, severe hypotension, and blood clotting, elucidating the biological targets in monocytes that could serve as potential biomarkers for the diagnosis, prognosis, and treatment of septic shock. We think that both YME1L1 and ECHDC3 may be the potential role in the mechanism of sepsis which may provide new insights into the pathophysiology of the disease.

In this study, we identified 4 biomarkers through gene expression analysis: YME1L1, ECHDC3, THEM4, and COQ10A. GSEA indicated that the 4 biomarkers were co-enriched in “oxidative phosphorylation” and other pathways, suggesting that these pathways may play important roles in sepsis. Based on the biomarkers, immunohistochemical analysis identified 13 cells with significant infiltration differences between sepsis and control samples and explored the correlation between differential immune cells and biomarkers. Finally, we analyzed the expression of biomarkers in clinical samples by RT-qPCR. All these analyses provide new insights into the diagnosis and treatment of sepsis. This study had several limitations. Firstly, the dataset related to sepsis and the sample size used for RT-qPCR experimental validation were insufficient. This was primarily due to multiple constraints including funding limitations, time restrictions, and difficulty in recruiting study subjects, which made it challenging to collect more samples. During the research design and data analysis processes, we implemented a series of measures to ensure the reliability and validity of the findings, such as hypothesis testing and correction methods. Secondly, due to insufficient funding and time, we were unable to conduct additional experimental validations, resulting in a lack of more direct supporting data. In the future, we will further validate and expand the current research outcomes through animal experiments, cell experiments, and other approaches such as immunofluorescence assay and western blot [50]. Lastly, the results required more measures in clinical actions to confirm its feasibility which is concerned with translational medicine.

## 5. Conclusion

Four biomarkers (YME1L1, ECHDC3, THEM4, and COQ10A) related to mitochondrial and macrophage polarization in sepsis were obtained by differential expression, machine learning, and expression validation. And they are co-enriched in the “oxidative phosphorylation” pathways. These findings provide targets for the early diagnosis of sepsis and the development of new methods for treatment.

## Data Availability

The datasets analyzed during the current study are available in the GEO database repository (https://www.ncbi.nlm.nih.gov/geo/), MitoCarta3.0 database repository (https://www.broadinstitute.org/mitocarta/mitocarta30-inventory-mammalian-mitochondrial-proteins-and-pathways), and MSigDB database repository (https://www.gsea-msigdb.org/gsea/msigdb).

## Supporting Information

Supporting Information 1 Supporting Table S1: specific clinical information for samples in the training set.

Supporting Information 2 Supporting Table S2: specific clinical information for samples in the validation set.

Supporting Information 3 Supporting Table S3: mitochondria-related genes (MCRGs) and macrophage polarization–related genes (MPRGs) were acquired from molecular signatures database.

Supporting Information 4 Supporting Table S4: specific clinical information for samples in RT-qPCR experiments.

Supporting Information 5 Supporting Table S5: details of sequences and primers in RT-qPCR experiments.

Supporting Information 6 Supporting Table S6: 44 GO terms included 12 protein hexamerization (BP), 2 mitochondrial inner membrane (CC), and 30 CoA carboxylase activity (MF).

Supporting Information 7 Supporting Table S7: 7 KEGG pathways encompassed “aminoacyl-tRNA biosynthesis,” “propanoate metabolism,” “selenocompound metabolism,” “fatty acid biosynthesis,” “fatty acid elongation,” “pyruvate metabolism,” and “valine, leucine, and isoleucine degradation.”

Supporting Information 8 Supporting Table S8: biomarker enrichment of the KEGG pathway.
